# Generating complex networks with time-to-control communities

**DOI:** 10.1371/journal.pone.0236753

**Published:** 2020-08-12

**Authors:** Guilherme Ramos, Sérgio Pequito

**Affiliations:** 1 Faculty of Engineering, University of Porto, Porto, Portugal; 2 Department of Industrial and Systems Engineering, Rensselaer Polytechnic Institute, Troy , NY, United States of America; Universidade de Lisboa Instituto Superior Tecnico, PORTUGAL

## Abstract

Dynamical networks are pervasive in a multitude of natural and human-made systems. Often, we seek to guarantee that their state is steered to the desired goal within a specified number of time steps. Different network topologies lead to implicit trade-offs between the minimum number of driven nodes and the time-to-control. In this study, we propose a generative model to create artificial dynamical networks with trade-offs similar to those of real networks. Remarkably, we show that several centrality and non-centrality measures are not necessary nor sufficient to explain the trade-offs, and as a consequence, commonly used generative models do not suffice to capture the dynamical properties under study. Therefore, we introduce the notion of time-to-control communities, that combine networks’ partitions and degree distributions, which is crucial for the proposed generative model. We believe that the proposed methodology is crucial when invoking generative models to investigate dynamical network properties across science and engineering applications. Lastly, we provide evidence that the proposed generative model can generate a variety of networks with statistically indiscernible trade-offs (i.e., the minimum number of driven nodes vs. the time-to-control) from those steaming from real networks (e.g., neural and social networks).

## Introduction

Unveiling the nature of dynamical and controllability properties of complex networks through their structure is key to the design of networks with desirable properties. These complex systems are present in nature, society, and technology. Examples include food webs in biological networks, the Internet, social networks, power systems, electrical circuits, nervous systems, as well as biochemical and biophysical systems [1].

In the last decade, we have witnessed an increasing understanding of the control properties of dynamical complex networks. Controllability ascertains that a network’s state can be attained within as much time as the network size. It is well-known that to guarantee the controllability of a network the minimum number of driven nodes is characterized to a certain extent by the degree distribution of the network [2, 3]. In fact, such characterization is at its core dependent of the disjoint family of cycles that cover all the nodes in the network, which in the limit leads to a dependency of the nodal dynamics [1]. Notwithstanding, the degree distribution is key in the characterization of the formation of the disjoint family of cycles that cover the networks nodes, and subsequently, the minimum number of driven nodes. Furthermore, it also impacts the other properties that lead to the characterization of the control profile to attain controllability [4].

As the networks grow bigger, it is of paramount importance to guarantee that a network can be steered to a desirable state in a relatively small amount of time. Specifically, it might not be possible to wait for as many time steps as the size of the network which is the requirement for controllability of the networks modeled as discrete-time linear time-invariant system (DT LTI) [5]. Therefore, it is fundamental to understand the dynamical and controllability properties of complex networks under time-to-control constraints. Trade-offs between the minimum number of driven nodes and the time-to-control were recently studied and captured by the notion of the *actuation spectrum* that maps the time-to-control to the minimum number of driven nodes [6, 7]. Intuitively, as we seek to steer the network’s state to a desirable goal within smaller time-windows, more driven nodes are required. For instance, if we need to control the network in a single time step, every single node in the network will need to be a driven node. As we allow more time-to-control, a smaller number of driven nodes is required.

Intriguingly, it has been empirically shown that some of the most commonly used generative models of artificial networks (e.g., small-world, scale-free, and Erdős-Rényi) fail to capture dynamical properties such as those described by the actuation spectra that resemble those found in real dynamical networks [7]. In the quest to overcome the limitations of current generative models, *we propose a generative model to create artificial dynamical networks with similar actuation spectra (i.e., similar trade-offs between the minimum number of driven nodes and the time-to-control) to those of real networks*.

Towards the aforementioned goal, we introduce the notion of *time-to-control communities* that combine networks’ partitions and degree distributions that is key for the proposed generative model. In fact, time-to-control communities are at the core of the proposed model, and enable to capture the required properties not captured by centrality and non-centrality measures, for which we provide empirical evidence hereafter. Therefore, the proposed study provides converging evidence that commonly used generative models do not capture the dynamical properties of real networks. As such, we believe that the proposed models are crucial when invoking generative models to investigate dynamical network properties across science and engineering applications. For instance, we provide evidence that the proposed generative model is able to generate a variety of networks with statistically indiscernible actuation spectra from those steaming from real networks (e.g., neural and social networks).

### Materials and methods

In what follows, we consider dynamical networks that are modeled by discrete-time linear time-invariant system (DT LTI):

$$\begin{array}{c} x(t+1)=Ax(t)+Bu(t),\;t=0,1,\ldots , \end{array}$$ (1)

where $x\left(t\right)\in R^{N}$ is the *state vector*, comprising the states of all network nodes at time *t*, *x*(0) = *x*_0_ is the initial vector state, and $u\left(t\right)\in R^{P}$ is the *input vector* of dimension *P* that is the signal injected to network at time *t*. The matrix $A\in R^{N\times N}$ is the state matrix and encodes the dynamic interdependences between nodes. The matrix $B\in R^{N\times P}$ is the input matrix and expresses which nodes are actuated by an external signal. Notice that applications entailing discrete-time dynamics include multi-agent systems, social networks, and communication systems, just to mention a few [8]. Furthermore, due to the digital nature of today’s controllers, signals are provided in a discrete fashion to the networks—some of which could be described as continuous-time dynamical models—that are ultimately discretized to perform analysis and control [9]. Thus, networks modeled as in (1) are pervasive in a multitude of natural and man-made systems, and, subsequently, the focus of our study.

Given a dynamical network described by matrices *A* and *B* as in (1), the *partial controllability matrix* of order *T* is defined as

$$\begin{array}{c} C\left(A,B;T\right)=\left[B\;AB\;\ldots ,A^{T-1}B\right]. \end{array}$$ (2)

In the case where *T* = *N*, the matrix C(A,B;N) is called the *controllability matrix*. A system (1) is *controllable* if for any initial state $x_{0}\in R^{N}$ and any desired state $x_{d}\in R^{N}$ there is an input signals ${\left\{u\left(t\right)\right\}}_{t=0}^{N-1}$ able to steer the system to *x*_*d*_, i.e., such that *x*(*T*) = *x*_*d*_. Furthermore, if the system is controllable it has to be so in at most *N* steps. Therefore, the notion of controllability implicitly assumes that we are given a time horizon of *T* = *N*. Thus, the system is controllable if and only if rank(C(A,B;N))=N [10].

In a plethora of complex network applications, not only is desired that the system is controllable, but also that we are able to steer the system to a desired state in a small window of time (much smaller than *N*). To deal with this specification, the concept of *controllability index* is key to our analysis [11], and it is defined as

$$\begin{array}{c} \tau (A,B)=\min\{T\;:\;rank(C(A,B;T))=N\}. \end{array}$$ (3)

Simply speaking, the smaller time-horizon for which there is a control law (i.e., a sequence of input signals) that ensure the system to be controllable. Nevertheless, in many contexts, it may not be possible to retrieve the dynamic interactions among network variables exactly. Instead, we can rely on the topology of the network. In other words, we may only have access to the location of their nonzero entries of the matrices A and B (i.e., the location of the edges in the network). In these circumstances, we can use *structural systems* theory tools to scrutinize the (necessary) controllability properties of almost all networks sharing the same topology that is commonly referred to as structural (or generic) controllability [12].

The structural controllability properties can be studied by investigating graph-theoretic properties of the *system digraph*, constructed by associating vertices to both state variables and input signals. Specifically, given the matrices in (1), if *A*_*ij*_ ≠ 0 then the system digraph has an edge from the state vertex *x*_*j*_ to the state vertex *x*_*i*_. Similarly, if *B*_*lm*_ ≠ 0 then the system digraph contains an edge from the input vertex *u*_*m*_ to the state vertex *x*_*l*_. In this case, the analog of the controllability index is the notion of *structural controllability index* [13, 14]. The structural controllability index of the system digraph of system (1) is the smallest *T* such that there are matrices *A*′ and *B*′ with $rank(C\left(A^{\prime },B^{\prime };T\right))=N$, with *A*′ and *B*′ having the same sparsity as *A* and *B*, respectively. Remarkably, invoking functional analysis arguments [15], it follows that almost all weighted networks associated with such system digraph can be controlled in, at least, *T* time steps. In other words, any random assignment of weights to the edges of the system digraph results (with high probability) in the same *time-to-control*.

Furthermore, the notion of structural controllability index enables us to assess the trade-off between the minimum number of *driven nodes* (i.e., the minimum number of state vertices that need to be actuated to ensure structural controllability) and the time required to steer a structural system to the desired state, which can be computed in polynomial time [1, 4]. The trade-offs between the number of driven nodes and the minimum time required to achieve an arbitrary network state can be captured by the notion of *actuation spectrum* of a network [6]. The actuation spectrum of a network given by an *N* × *N* matrix *A* is defined as the sequence of integers ${\left\{s\left(A,T\right)\right\}}_{T=1}^{N}$, where *s*(*A*, *T*) = *n*_*T*_ is the minimum number of driven nodes required to actuate the network and that yield a controllability index of *T*. Note that the actuation spectrum is tied with energy budget in the sense that as we allow more time for the system to be controlled, less energy would be required as the controllability Gramian is monotonically decreasing with the time horizon [16].

Now, in order to derive the generative model to attain a specified actuation spectrum, consider the decomposition of a network in the smallest number of non-overlapping (weakly connected) subnetworks (i.e., *partitions*) with at most *k* nodes. It is easy to see that by ensuring controllability for each subnetwork, we can guarantee that the entire network is controllable in at most *k* time steps [7]. Furthermore, the minimum number of driven nodes to control the network in *k* time steps corresponds to the sum of the minimum number of driven nodes from the different subnetworks, across all possible partitions [7].

**Fig 1:**
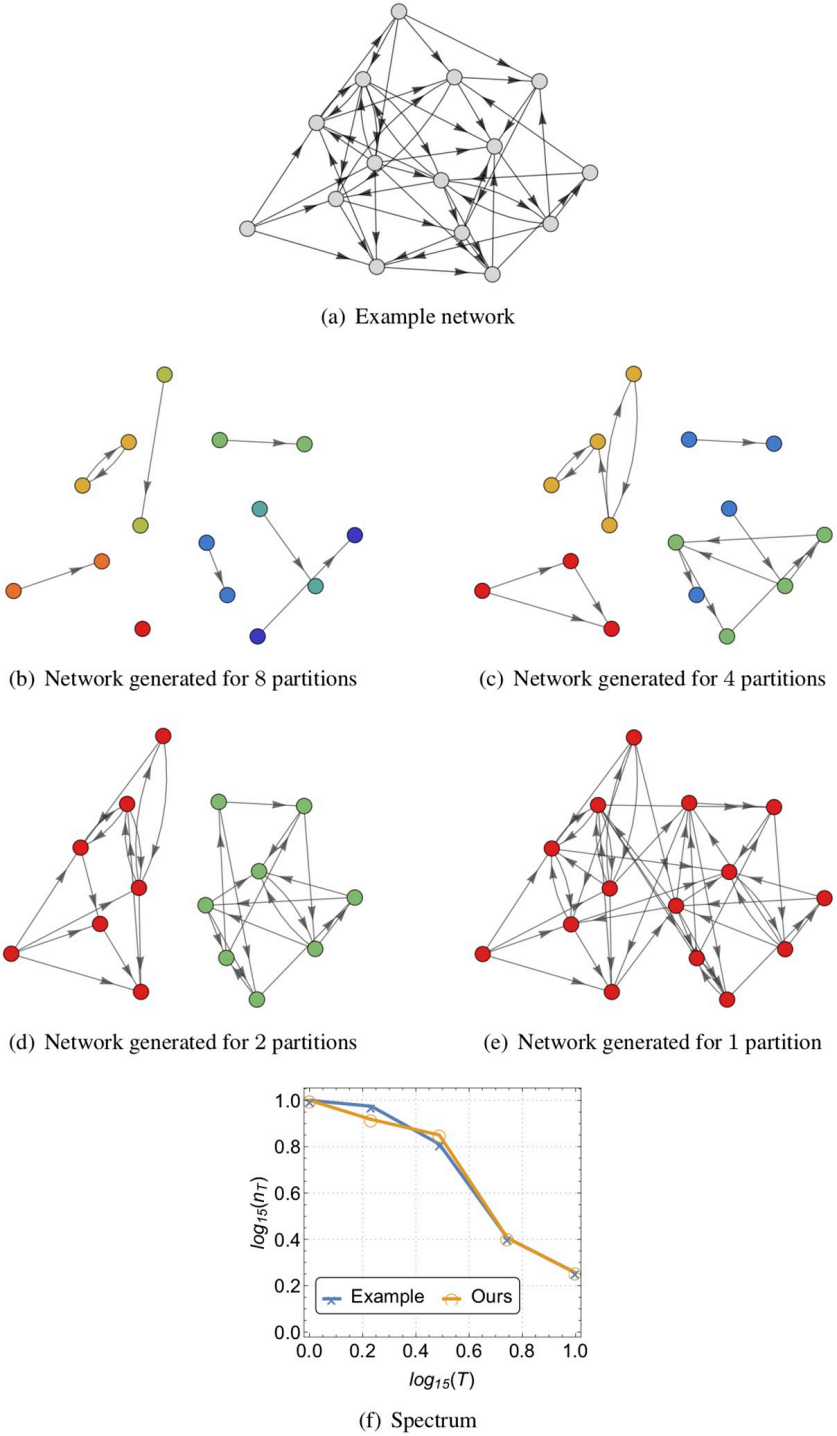
Illustrative example of Algorithm 1 with 8, 4, 2 and 1 partitions. (a) depicts an example network with 15. In (b), we consider 8 partitions, where by ensuring the structural controllability with the minimum number of driven nodes for each subnetwork, we attain structural controllability of the entire network with a controllability index of 2. Similar reasoning applies to 4 partitions (c), 2 partitions (d), and 1 partition (the entire network depicted in (e)), yielding the statistically indiscernible actuation spectrum presented in (f).

In the same way controllability of a network is dictated by the degree distribution, at the subnetwork level the minimum number of driven nodes is also dictated by the degree distribution. Thus, a *time-to-control community of size k* corresponds to collection of the subnetworks ${\left\{N_{i}^{k}\right\}}_{i=1}^{n_{k}}$ on the *n*_*k*_ partitions of size at most *k*, and the corresponding in- and out-degree distributions ${\left\{\left(d_{k,i}^{-},d_{k,i}^{+}\right)\right\}}_{i=1}^{n_{k}}$. Interestingly, the time-to-control community seem to play the role of controllability ‘network motifs’ [17] since, for increasing *k*′ > *k*, the degree distributions for the time-to-control community of size *k*′ builds upon those of size *k* over, possibly overlapping, partitions between different time-to-control communities. In particular, by imposing a specific in- and out-degree distributions of the time-to-control communities at time-to-control communities with lower size, there is an underlying structure imposed on the in- and out-degree distributions of the time-to-control communities with increasing size—see Fig 1.

That said, we propose a generative model that produces networks with desirable trade-offs between time-to-control and the minimum number of driven nodes (i.e., actuation spectrum) by leveraging key ideas from hierarchical bootstrapping [18]. Given ${\left\{N_{i}^{k},\left(d_{k,i}^{-},d_{k,i}^{+}\right)\right\}}_{i=1}^{n_{k}}$ describing time-to-control communities of size *k* for *k* = 1, …, *n*, with *n* the dimension of the network of interest, we can proceed iteratively as follows: for increasing values of *k* ∈ {1, …, *n*}, we generate an in- and out-degree distribution of each subnetwork ${\left\{N_{i}^{k}\right\}}_{i=1}^{n_{k}}$ by considering the degree distributions of the subnetworks associated with time-to-control communities of size smaller than *k*. Specifically, we consider $\left(d_{k,i}^{-}-{\bar{d}}_{i;1:k-1}^{-},d_{k,i}^{+}-{\bar{d}}_{i;1:k-1}^{+}\right){\}}_{i=1}^{n_{k}}$, where ${\bar{d}}_{i;1:k-1}^{-}$ and ${\bar{d}}_{i;1:k-1}^{+}$ are the in- and out-degrees of the subnetwork *i* generated at previous iterations, and generated using, for instance, the Chung-Lu method [19, 20]—the method is summarized in Algorithm 1. Note that the proposed model does not require that the obtained network yields the same actuation spectra as the original network, but it allows to achieve a statistically indistinguishable actuation spectra. We use the Komolgorov-Smirnoff (KS) test to assess if we cannot reject the null hypothesis that two distributions have the same cumulative distribution function, by considering a significance level of 5%.

In Fig 1, we provide an illustrative example of Algorithm 1. Specifically, consider a network with 15 nodes depicted in Fig 1(a), which actuation spectrum is provided in Fig 1(f). For an increasing time-to-control community (i.e., decreasing number of partitions), we sample the degree distribution of the corresponding subnetworks of the original network determined by the partitions considered. In Fig 1, we illustrate the latter procedure with 8, 4, 2, and 1 partitions as illustrated in Fig 1(b)–1(e), respectively. In particular, when 8 partitions are considered (i.e., Fig 1(b)), by ensuring the structural controllability with the minimum number of driven nodes for each the subnetwork, we attain structural controllability of the entire network with a controllability index of 2. We can invoke a similar reasoning for 4 partitions (i.e., Fig 1(c)), 2 partitions (i.e., Fig 1(d)), and 1 partition (i.e., the entire network depicted in Fig 1(e)), which leads to the actuation spectrum in Fig 1(f). We notice that both actuation spectra (i.e., the initial network and the one generated with the proposed model) are statistically indiscernible (Komolgorov-Smirnoff (KS) test with significance level of 0.05).

**Algorithm 1** Progressive Partitions Degree Distribution

**Input:** Network G=(V,E) and a set P with partition sizes

**Output:** Network H with the same degree distribution as G

1: $H=(V^{\prime },E^{\prime })$ with *V*′ = *V* and *E*′ = ∅

2: **for each** i∈P

3:  S=partitions(G,i)

4:  **for each** V∈S

5:   $G_{V}=\text{subgraph}\left(G,V\right)$

6:   $H_{V}=\text{subgraph}\left(H,V\right)$

7:   $id=\text{indegree}(G_{V})-\text{indegree}\left(H_{V}\right)$

8:   $od=\text{outdegree}(G_{V})-\text{outdegree}\left(H_{V}\right)$

9:   *E*′ = *E*′∪ edges(randomGraph(*id*, *od*))

10: $H=(V^{\prime },E^{\prime })$

**Fig 2:**
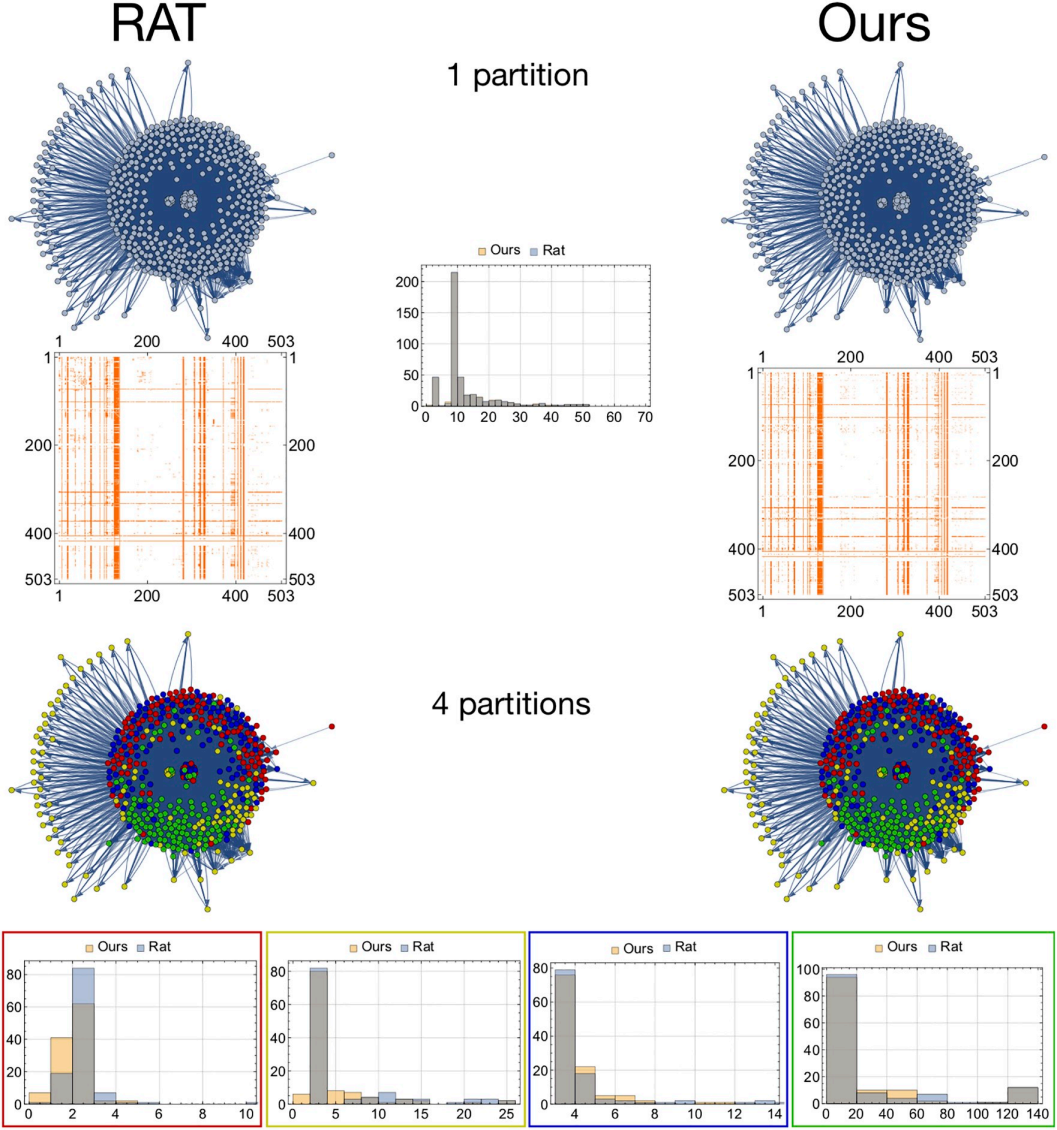
Degree distributions across different subnetworks with equal sized partitions for the rat structural connectivity network and the one generated by our model considering partitions with *k* nodes, with *k* ∈ {5, 25, 50, 100, 200, 300, 503}.

Lastly, we notice that, in step 3 of Algorithm 1, we do not prescribe any specific partition sizes, and there could be several possible partitions. Nonetheless, as previously mentioned, due to the overlapping nature of the increasing size partitions, the degree distributions get constrained leading to overall degree distributions of the generated networks that are statistically indiscernible from the original network. Additionally, some of the issues reported by the nodal dynamics previously reported [1], can also be circumvented as we can consider partitions of size 1, which will lead to the replication of self-loops in the generated network if they existed in the original network.

In what follows, we consider the following real networks: (neural networks) *Rattus norvegicus* (Rat) with 503 neurons, *C. elegans* with 277 neurons, and macaque with 95 regions [21–24]; (social network) high-school [25]; (trophic) Florida ED [26]; (combinatorial problem) 130 Bit [27]; and (animal dominance) Bison [28]. The network characteristics are presented in Table 1. We would like to observe that we selected networks across different science and engineering applications that have been commonly invoked to study controllability properties. For example, the neural networks could be seen as steering the brain activity towards a cognitive/task profile within a given time. Alternatively, social networks could be executing a dynamic agreement protocol where the state nodes represent the agent’s opinion that we now seek to steer to a desirable opinion profile within a given amount of time, while using the minimum number of “influencers” (i.e., driven nodes).

**Table 1:** Details of the networks considered in the current study.

	Rat [24]	C. elegans [21]	Macaque 95 [22]	Highschool [25]	Florida ED [26]	130 bit [27]	Bison [28]
Nodes	503	277	95	70	128	584	26
Edges	27,667	2,105	2,390	366	2,137	6,120	314

## Results

We seek to obtain generated networks which actuation spectra (i.e., trade-offs between time-to-control and a minimum number of driven nodes) is similar (i.e., statistically indiscernible) to the actuation spectra of the real networks described in Table 1. Towards this goal, we will deploy the methodology proposed in the Materials and Methods section. To avoid over-specification of time-to-control communities, we consider just a few as described next.

**Fig 3:**
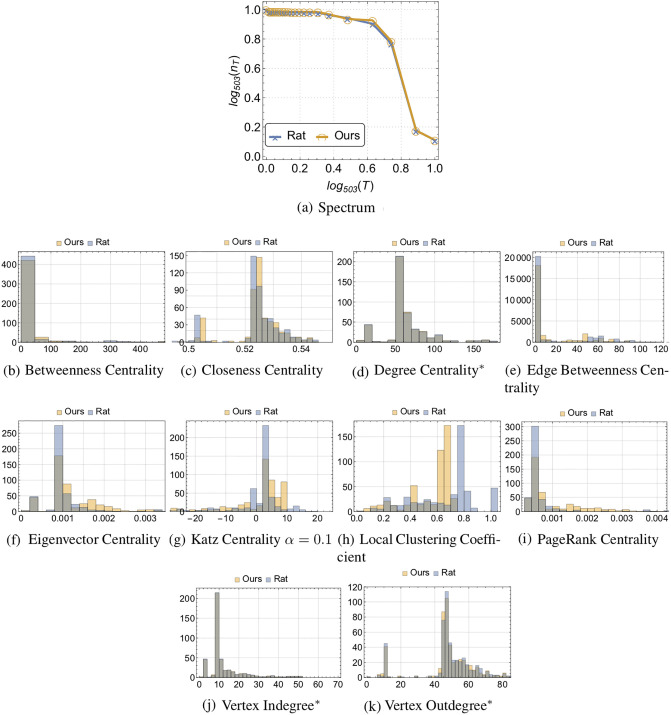
Different centrality and non-centrality measures and degree distributions (where the asterisk denotes the statistically indiscernible distributions using KS test with significance level of 0.05), and actuation spectrum (i.e., tradeoffs between minimum number of driven nodes and time-to-control) across different subnetworks with equal sized partitions for the rat structural connectivity network and the one generated by our model considering partitions with *k* nodes, with *k* ∈ {5, 25, 50, 100, 200, 300, 503}.

First, we consider the neural networks for the rat and the C. elegans. The brain regions can be specified by different atlas (i.e., size and number of regions considered informed by the underlying anatomy or function). The white matter crisscrossing the different regions define the connectivity often refer to as structural connectivity of the brain that crafts the brain dynamics [29, 30]. There is evidence that structural connectivity networks exhibit small-world properties [31], but there is also evidence that such models fail to capture the tradeoffs between the minimum number of driven nodes and time-to-control [7]. In Fig 2, we compare the structural connectivity matrices of the rat’s brain with 503 regions and the one generated by our model by considering only time-to-control communities of size *k* ∈ {5, 25, 50, 100, 200, 300, 503}. Remarkably, we were able to retrieve a statistically indistinguishable actuation spectrum (KS test with significance level of 0.05). Additionally, we present different degree distributions across different subnetworks that consider one and four partitions that are statistical significantly different (KS test with a significance level of 0.05). Notwithstanding, at the network level (i.e., with one partition), the degree distributions are statistically indiscernible, under the same non-parametric statistical test—see Fig 3(j) and 3(k). Remarkably, different centrality and non-centrality measures (specifically, the betweenness centrality, closeness centrality, edge betweenness centrality, eigenvector centrality, Katz centrality, local clustering, and PageRank) presented in Fig 3(b)–3(k) are statistically different (KS test with a significance level of 0.05).

Another typical example of neural network is the C. elegans where each vertex represents a neuron and the structure is capture by the axon connections between different neurons [21]. We considered time-to-control communities of size *k* ∈ {2, 4, 5, 10, 20, 40, 100, 277}, and similarly to the structural connectivity networks of the rat and macaque, we were able to recreate a statistically indiscernible actuation spectrum while we can observe that only the centrality degree, in- and out-degree are statistically indiscernible (KS test with a significance level of 0.05)—see Tables 2 and 3. In other words, the remaining centrality and non-centrality measures considered are statistically different between the original and generated network despite the statistically indiscernible actuation spectrum.

Lastly, for the sake of completeness, we have considered a variety of networks from different science and engineering applications—see Table 1. Specifically, we consider the following time-to-control communities indexed by *k* for the proposed generative model:

(*i*) *Macaque 95* which comparison between the original network and the generated one by the proposed approach with *k* ∈ {10, 20, 40, 95}; (*ii*) *Highschool* with *k* ∈ {2, 3, 4, 8, 20, 40, 70}; (*iii*) *Florida ecosystem dry* with *k* ∈ {5, 20, 50, 128}; (*iv*) *130 bit* with *k* ∈ {2, 4, 8, 10, 20, 50, 100, 292, 584}; and (*v*) *Bison* with *k* ∈ {2, 3, 4, 6, 13, 26}. The comparison of centrality and non-centrality measures between the original and generated networks (with statistically indiscernible actuation spectra) is reported in Tables 2 and 3, respectively.

**Table 2:** Centrality measures, the asterisk denotes the statistically indiscernible distributions using KS test with significance level of 0.05.

	Betweenness Centrality	Closeness Centrality	Degree Centrality	Edge Betweenness Centrality	Eigenvector Centrality	Katz Centrality	PageRank Centrality
Rat			*				
C. elegans	*		*	*			
Macaque 95			*				
Highschool	*		*			*	*
Florida ED			*				*
130 bit			*				
Bison	*	*	*	*	*		*

**Table 3:** Non-centrality measures, the asterisk denotes the statistically indiscernible distributions using KS test with significance level of 0.05.

	Local Clustering Coefficient	Vertex Indegree	Vertex Outdegree
Rat		*	*
C. elegans	*	*	*
Macaque 95		*	*
Highschool		*	*
Florida ED		*	*
130 bit		*	*
Bison	*	*	*

## Discussion of results

The proposed methodology allows us to generate networks with similar actuation spectra (i.e., trade-offs between the time-to-control and the minimum number of driven nodes) similar to those of real-networks. We provided evidence that current centrality and non-centrality measures are unable to justify these trade-offs; namely, betweenness centrality, closeness centrality, edge betweenness centrality, eigenvector centrality, Katz centrality, local clustering, and PageRank. As a consequence, current generative models are unable to synthesize networks with dynamical properties similar to those capture by the actuation spectrum. As such, our methodology provides the first generative model to generate networks with a specific actuation spectrum. Therefore, we believe that the proposed generative model will enable us to unveil new insights on how the structure crafts the networks’ dynamics, and it equips us with a methodology towards the design (e.g., re-wire) networks to attain desirable time-to-control properties that mimic real-world networks. Thus, with an impact in analyzing and designing networks in the context of both science and engineering applications.

It is worth noticing that some variability is expected across the actuation spectra as a consequence of both the partitions obtained and degree distribution generative methods. Specifically, due to the non-uniqueness of partitions, we have randomized the labels of the nodes towards obtaining different partitions, which led to similar results. Also, we have performed several samplings of the degree distributions without a significant change in the results attained. On the one hand, the problem of finding the minimum number of partitions of a graph with at most *k* nodes is NP-hard. The methods considered are approximation schemes that have shown experimentally errors of approximately 4% on average [32]. On the other hand, generative models to obtain desirable degree distributions are known to provide only expected degree distributions with lower variance as the number of nodes goes to infinity, which implies that some variability is expected for networks with small dimensions.
